# Regulation of RSPO3-LGR4 Signaling: Emerging Role in Inflammation Revealed by Network Analysis

**DOI:** 10.26502/acbr.50170489

**Published:** 2025-10-27

**Authors:** Marija Stojanovic, Devendra K. Agrawal

**Affiliations:** 1 Department of Translational Research, Western University of Health Sciences, Pomona, California 91766, USA; 2 Institute of Medical Physiology “Richard Burian”, Faculty of Medicine, University of Belgrade, 11000 Belgrade, Serbia

**Keywords:** RSPO3, LGR4, Wnt signaling, Upstream regulation, Downstream regulation, Kinases, Inflammation, Cytokines, Transcription factors, microRNAs, *In silico* analysis

## Abstract

RSPO3, as a member of the R-spondin gene family, is a secreted molecule that enhances one of the fundamental biological pathways, the canonical Wnt signaling pathway. Once secreted from endothelial cells or macrophages, it typically binds to specific receptors from the LGR family. Additionally, LGR-independent activation of the Wnt cascade, driven by RSPOs, has also been described, mediated by heparan sulfate proteoglycans (HSPGs). LGR4 (GPR48) belongs to the G-protein-coupled receptor superfamily, subfamily B. It is widely recognized as an RSPO3-binding receptor and, thereafter, a Wnt signaling potentiator. Expression patterns of both RSPO3 and LGR4 have been found across various tissues. RSPO3 regulates stem cell maintenance in the intestine, in addition to its function in liver endothelial cells in terms of liver zonation or osteoblast differentiation. LGR4 has shown expression in hypothalamic neurons, regulating reproductive hormone secretion and control of food intake. Various studies reported the contribution of both RSPO3 and LGR4 to inflammatory cascades. Specifically, the RSPO3-LGR4 ligand-receptor interaction was shown to activate the NLRP3 inflammasome and β-catenin-NF-kB signaling cascade. Endothelial-derived RSPO3 exerts regenerative potential via the RSPO3-LGR4-ILK-AKT pathway, as presented *in vitro* model of inflammatory vascular injury. As a reaction to H.pylori infection, NF-κB was produced in response to RSPO3-LGR4 interaction. In order to get better insight into the signaling cascade between RSPO3 as a ligand and LGR4 as a receptor, in the context of inflammation, in silico analysis was performed. Gene input list included core Wnt pathway proteins, their downstream molecules, and various inflammatory mediators and cytokines related to RSPO3 and LGR4, as described in the literature. Network analysis included protein-protein, transcription factor-gene, and microRNAs-gene interactions. Molecules revealed from the network analysis are potential therapeutic targets in the treatment of inflammatory conditions. Further investigations are needed to test the predicted molecular pathways *in vitro* or *in vivo*. From the translational point of view, providing a proper anti-inflammatory agent in the clinical setting will be the ultimate research goal.

## Introduction

R-spondins (roof plate-specific spondins) are a group of cysteine-rich, secreted glycoproteins that regulate numerous cellular processes and signaling pathways [1]. High structural and approximately 60% sequence homology have been identified in four members of the R-spondin family (RSPO1-RSPO4). R-spondin3 (RSPO3) was identified in 2002 as the first member of the R-spondin gene family [2]. It was initially referred to as PWTSR, recognized for encoding a novel human protein containing a thrombospondin type 1 repeat (TSR) domain [2]. Four domains have been identified in R-spondins: cysteine-rich furin-like (FU1, FU2) domains, a putative signal peptide domain, a thrombospondin (TSP) type I repeat domain, and a basic amino acid-rich (BR) domain [3]. The FU1, FU2, and TSP/BR domains of RSPO proteins facilitate their interaction with several key molecules, including the E3 ubiquitin ligases ZNRF3 and RNF43, the leucine-rich repeat-containing G protein-coupled receptors (LGR4 to LGR6), and heparan sulfate proteoglycans (HSPGs) [4]. Activation of canonical Wnt signaling, one of the crucial pathways for cell growth and tissue development, has been shown to be enhanced by the FU domain. It has been shown that R-spondins mediate Wnt-signaling via Leucine-rich repeat G-protein-coupled receptors (LGR receptors). Herein, it is worth mentioning that Wnt potentiation by R-spondins is possible without LGRs, in the presence of and mediated by HSPGs [5]. Taking all the above-mentioned into account, R-spondins play a pivotal role as important regulators of tissue homeostasis [6].

From a physiological perspective, RSPO3 is involved in regulating the cardiovascular system, liver and gut function, as well as skeletal muscles and bone homeostasis [7–10]. In fact, it is the key regulator of cardiac development and vascular stability [11]. Furthermore, R-spondin3 effects in the heart are mainly mediated through Lgr4 receptor [12]. Additionally, recent research in mice suggests that secretory protein RSPO3 activates the Wnt signaling pathway, thereby contributing to the restoration of stem cell function and the regeneration of colon epithelial tissue [13]. Selective expression of RSPO3 was found in the endothelial cells of the liver’s central vein, playing a crucial role in preserving the metabolic zonation of the liver [14]. Recent mechanistic studies suggest that RSPO3 positively influences osteoblast proliferation and differentiation, promoting these processes more effectively [15,16]. Furthermore, studies in mouse models have confirmed that excessive RSPO3 activity can directly promote tumor formation in the intestinal epithelium, suggesting its oncogenic potential [17].

Leucine-rich repeat (LRR)-containing G-protein-coupled receptor 4 (LGR4) or G-protein- coupled receptor 48 (GPR48) belongs to the GPCR superfamily, subfamily B [18]. In addition to LGR4, there are two other members of GPCR subfamily B: LGR5 and LGR6. All three members share significant sequence homology: 46% between LGR4 and LGR5 and 44% between LGR4 and LGR6 [19]. The common expression of LGR4 has been found in various human tissues, including the ovary, kidney, heart, gastroesophagus, and pancreas [20]. LGR4 is well known as an intestinal crypt stem cell marker. In addition to high LGR4 expression in Paneth cells, it has been shown that Paneth cells differentiation depends on sufficient LGR4 expression [21,22].

LGR4 also regulates the development of the eye and reproductive system as a response to its interaction with RSPOs and subsequent activation of Wnt signaling [23]. Herein, it is worth mentioning that LGR4 is known as Wnt signaling facilitator, since it enhances Wnt signaling cascade by interacting with specific ligands (R-spondins). This type of interaction between R-spondins and LGRs’ extracellular domain was described for the first time in 2011, reporting LGR4 and LGR5 as receptor molecules [24,25]. In the context of canonical Wnt signaling, LGR4 has been identified as a key contributor to skeletal system development, regulating osteogenesis during both the embryonic and postnatal period [26]. Another study showed the role of LGR4 in puberty regulation by controlling the function of hypothalamic gonadotropin-releasing hormone (GnRH) neurons, essential for the regulation of pituitary-derived reproductive hormones [27]. Some other functions of the hypothalamus have also been shown to be regulated by LGR4, as well [28]. Namely, RSPO3 expression has been identified in the hypothalamic neurons that control food intake [29]. Therefore, it has been reported that intracerebroventricular application of RSPO3 leads to inhibition of food intake, as a result of binding to LGR4 [25]. Furthermore, LGR4 overexpression has been associated with poor patient outcomes in breast and ovarian cancers [30,31].

Despite the various above-mentioned biological functions that RSPO3 and LGR4 contribute to, their role in inflammation and oncogenesis has been reported across various tissues (stomach, intestine, lungs, heart, blood vessels). It is of great importance, if we consider the fact that inflammation is a hallmark of some chronic diseases such as cancer, cardiovascular diseases, diabetes, gastrointestinal, respiratory or neurological disorders [32,34]. Modulation of RSPO3 and LGR4 expression in inflammatory conditions might be beneficial for the control of disease progression. General reaction to pro-inflammatory stimuli includes cellular cytokine receptors activation (IL1, toll like receptors-TLR, IL6) leading to further stimulation of kinases [35,36]. Nevertheless, cellular mechanisms of RSPO3 and LGR4 signaling in response to inflammation are yet to be elucidated.

Considering the diverse roles of RSPO3 and LGR4 in embryonic tissue development and stem cell regulation under physiological conditions, this article aims to explore the RSPO3-LGR4 interaction in both physiological and pathological contexts, particularly inflammation. Despite all the above-mentioned, the molecular interactions of RSPO3–LGR4 signaling in the context of inflammation remain incompletely understood. Therefore, we performed network analysis to identify potential mediators (transcription factors, miRNAs) involved in the regulation of this signaling axis in more detail.

### R-SPONDIN 3 Signaling and Regulation

The upstream regulators of RSPO3 vary by tissue and biological context (tissue development, inflammation, cancer). RSPO3 is regulated transcriptionally and post-transcriptionally, mechanically, and epigenetically by a range of signaling pathways. Some key upstream and downstream regulators of RSPO3 identified in different biological systems are mentioned in the following text.

### Upstream regulation of RSPO3 expression

In the context of cardiac tissue development, transcription factor Nkx2–5 (NK2 Homeobox 5) has been shown to modulate Wnt signaling by regulating RSPO3 expression [11]. Nkx2–5 is a cardiac-specific transcription factor identified as essential for the specification and proliferation of cardiac cells [37]. By upregulating RSPO3, it participates in ongoing cardiac growth during tissue development. Transcriptional activation of RSPO3 by FOXC1 (Forkhead box protein C1) and FOXC2 (Forkhead box protein C2) has been shown in lymphatic endothelial cells located in pericryptal lymphatic vessels, regulating lymphangiogenesis [38]. Recently, RSPO3 has been reported as a contraction-inducible factor acting as a paracrine myokine supporting myogenesis, as shown in an *in vitro* exercise model [9]. According to this study, RSPO3 was upregulated in response to muscle contractile activity. Inflammation contributes to RSPO3 expression as well, since RSPO3 was found to be upregulated following Helicobacter Pylori infection, promoting gastric stem cells proliferation and hyperplasia [39]. Furthermore, genetic alterations, such as the PTPRK-RSPO3 gene fusion, can result in elevated RSPO3 expression, contributing to colorectal tumorigenesis [40]. Epigenetic modifications also regulate RSPO3 oncogenic expression. In fact, targeted promoter DNA demethylation leading to RSPO3 upregulation has been shown to suppress cholangiocarcinoma progression [41]. Endocrine control of RSPO3 expression has been described as well. Namely, treatment with estradiol (E2) in an experimental model of ovariectomized mice showed increased bone expression of RSPO3 [42].

### Downstream regulation of RSPO3 expression

Downstream regulation of RSPO3 expression primarily involves activation of Wnt/β-catenin signaling. In addition to this pivotal role, RSPO3 regulates other pathways depending on the tissue-specific functions.

As previously mentioned, Wnt/β-catenin pathway is essential for tissue development and regeneration. Since the beginning of Wnt signaling research in 1982, when the first member of the Wnt family was identified, research in this field has significantly risen [43]. Therefore, it has been already reported that Wnt/β-catenin pathway participates in different pathophysiological mechanisms, including both non-cancer (atherosclerosis, bone pathology, wound healing, hair loss, neurodegeneration) and cancer diseases (colorectal, hepatocellular and breast cancer) [44–48]. Wnt signaling pathway typically includes canonical (β-catenin dependent) and non-canonical (β-catenin independent) signal transduction [49].

The canonical pathway is activated by specific Wnt ligands, including Wnt2, Wnt3, Wnt3a, and Wnt8a. Initially, Wnt binds to two types of membrane receptors called Frizzled (Fzd) receptor and LDL-receptor-related proteins 5 and 6 (LRP5 and LRP6) [50]. Subsequently, Wnt-Fzd-LRP5/6 complex-mediated recruitment of scaffolding protein Dishevelled (DVL) and Axin will lead to inhibition of β-catenin phosphorylation and its stabilization [51]. The hallmark of the canonical Wnt pathway is translocation of β-catenin from the cytoplasm to the nucleus, where it enables T-cell factor/lymphoid enhancer factor (TCF/LEF) transcription factors, leading to specific target gene expression [52]. Counterpart, in the absence of Wnt, β-catenin undergoes sequential phosphorylation, marking it for recognition by the SCFβ-TRCP ubiquitin ligase complex, which facilitates its ubiquitination and subsequent degradation by the proteasome [53,54]. Phosphorylation of β-catenin takes place within a multiprotein destruction complex composed of key components such as Axin, GSK3β (Glycogen synthase kinase-3 beta), the tumor suppressor APC (adenomatous polyposis coli), and CKI (casein kinase I), which is recruited to the complex by Diversin. Axin and APC serve as scaffolding proteins that bring β-catenin into proximity with GSK3β, facilitating its phosphorylation. Additionally, GSK3β can phosphorylate Axin and APC themselves. This phosphorylation process ensures that cytoplasmic β-catenin levels remain low, thereby preventing its translocation to the nucleus and subsequent interaction with TCF/LEF transcription factors. In the absence of Wnt signaling, TCF/LEF proteins in the nucleus are bound by a family of corepressors known as Groucho (Grg) proteins, which maintain repression of Wnt target gene transcription [55,56]. The Wnt/β-catenin-independent signaling was shown to mediate the effects of RSPO3 on the proliferation of limbal epithelial stem cells (LESCs) [57]. Furthermore, RSPO3 was shown to be an antagonist to bone morphogenetic protein receptor 1A (BMPR1A) in an early embryonic development model [58].

Herein, it is worth mentioning that the non-canonical Wnt pathway typically includes two signaling cascades: Wnt/Ca^2+^ pathway and the PCP (Planar Cell Polarity) pathway [59]. Elevated intracellular concentration of Ca^2+^ following Wnt/Ca^2+^ activation, leads to calcineurin activation. Calcineurin, as a phosphatase, dephosphorylates the transcription factor nuclear factor of activated T cells (NFAT) [60]. In this context, it has been shown that RSPO3 controls vascular stability through Non-canonical WNT/Ca^2+^/NFAT signaling pathway in endothelial cells [61].

### RSPO-LGR signaling and downstream pathways regulation

It is of great importance to emphasize interaction between R-spondins and LGRs, as the RSPO–LGR axis represents a key ligand–receptor system involved in the regulation of the Wnt signaling pathway.

**Figure 1:**
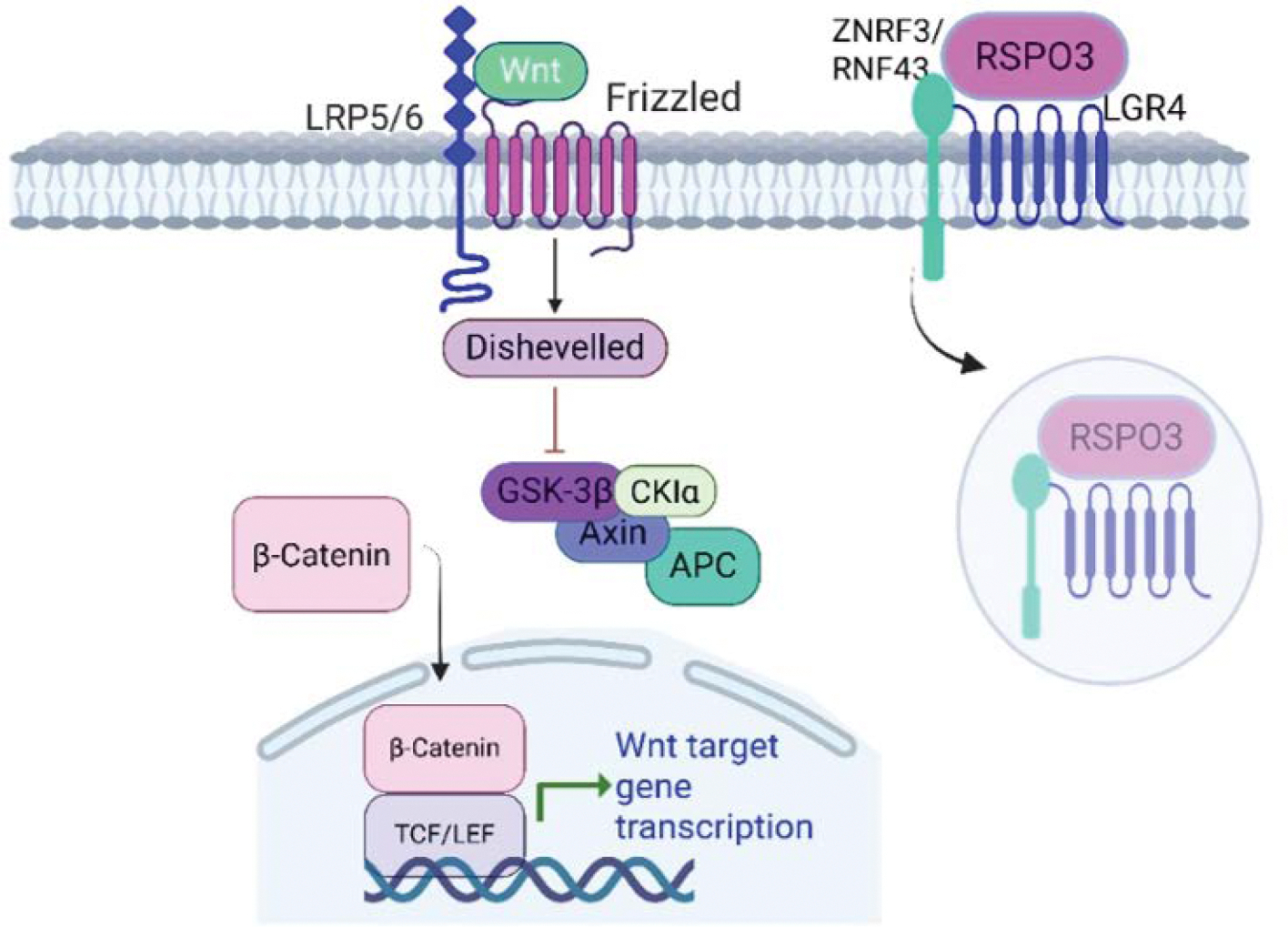
RSPO3 binds to LGR4 and potentiates Wnt/β-catenin signaling through Frizzled receptors. RSPO3-LGR4 binding promotes ZNRF3/RNF43 autoubiquitination and membrane clearance. Once the ligases are removed from the membrane, more Wnt ligands become available to Frizzled receptors, which enhance the signaling pathway of β-catenin production. LRP5/6 (low-density lipoprotein receptor-related protein 5/6); GSK3β (Glycogen synthase kinase-3 beta); APC (adenomatous polyposis coli); CKI (casein kinase I); TCF/LEF (T-cell factor/lymphoid enhancer factor); ZNRF3 (zinc and ring finger 3); RNF43 (ring finger 43).

RSPO3 acts as a potent enhancer of Wnt/β-catenin signaling by binding to LGR4, LGR5, or LGR6 receptors. Meaning, it does not activate the Wnt signaling cascade directly. The interaction of RSPO3 with LGR receptors leads to the inhibition of the E3 ubiquitin ligases zinc and ring finger 3 (ZNRF3) and ring finger 43 (RNF43), which normally promote the degradation of Frizzled and LRP5/6 receptors, ultimately resulting in the stabilization of these receptors. When stable, they can further increase cellular sensitivity to Wnt ligands, leading to activation of the cascade and cellular accumulation of β-catenin [62–64] (Figure 1).

In addition, it has been shown that RSPO3 regulates canonical Wnt signaling in bones by two distinct pathways, including LGR/RNF43/ZNRF3 and LGR/extracellular signal-regulated kinase 1 (Erk) axes. According to this study, RSPO3 stimulation of the LGR/RNF43/ZNRF3 cascade leads to β-catenin stabilization and activation of Wnt signaling. On the other hand, it was proposed that stabilization of β-catenin was prevented mediated by RSPO3/LGR/Erk axis through impaired Erk signaling, suggesting a role of RSPO3/Erk/Wnt pathway in regulation of bone homeostasis [10]. Another *in vitro* study on human adipose stem cells suggests that activation of Erk signaling upon RSPO3 silencing is mediated by LGR4 via the Erk/fibroblast growth factor (FGF) axis [65].

The intracellular scaffold protein IQ motif-containing GTPase-activating protein 1 (IQGAP1) is identified as a scaffolding protein, meaning it mediates interactions between molecules and signaling pathways, linking them together, and therefore regulates various cellular processes like cell adhesion, migration, actin cytoskeleton organization, or cell cycling [66]. IQGAP1 has been found to mediate RSPO-LGR4 interaction with the Wnt pathway, as well. Namely, upon RSPO stimulation, LGR4 interacts with IQGAP1 leading to IQGAP1-DVL association. Therefore, the RSPO-LGR4-IQGAP1 axis was shown to enhance both β-catenin-dependent (phosphorylation of LRP5/6 via MEK1/2 (MAP/ERK kinase1/2) and β-catenin-independent pathways (actin filament regulation) [67]. RSPO3-LGR4 interaction has been found to exert neuroprotective effects, as shown in the ischemia-reperfusion *in vitro* model. According to this study, Erk was reported as the downstream effector kinase in the LGR4-G protein subunit alpha i1/3 (Gαi1/3)-GRB2-associated binding protein 1 (Gab1)-Erk (LGR4-Gαi1/3-Gab1-Erk) axis, activated by RSPO3 [68].

### Downstream Regulation of LGR4 Expression

It has been demonstrated that LGR4 regulates cellular processes beyond the Wnt signaling pathway, as well. In the context of bone formation, LGR4 regulates osteoblast function through a cascade of cyclic adenosine monophosphate (cAMP)-protein kinase A (PKA)-cAMP response element-binding protein (CREB), subsequently leading to control of Activating Transcription Factor 4 (Atf4) and its target proteins (collagen) and genes that controls formation of non-collagenous proteins of the bone matrix such as osteocalcin (OCN) and bone sialoprotein (BSP) [26]. Osteoclastogenesis is inhibited by the binding of Ligand of receptor-activator of NFκB (RANKL) to LGR4. RANKL is one of the few ligands found to bind to LGR4, likewise RSPOs, Norrin, or Nidogen-2 (Zhang, 2023). After binding to LGR4, RANKL stabilizes Glycogen Synthase Kinase 3 Beta (GSK3β), leading to its activation, and subsequently phosphorylation of Nuclear Factor of Activated T-Cells (NFATC), which, once phosphorylated, does not enter the nucleus, thus inhibiting osteoclastogenesis [29].

### RSPO3 and LGR4 Signaling related to Inflammation

Although existing literature supports the involvement of RSPO3 and LGR4 in inflammation across various tissues, the precise underlying mechanisms remain to be fully elucidated. There is a growing body of evidence indicating the role of RSPO3 in inflammatory processes, which exhibits both proinflammatory and anti-inflammatory actions [70,71], Similarly, LGR4 signaling was shown to regulate inflammation positively and negatively (72, 73). We are particularly interested in reviewing literature from various experimental models that explore the RSPO3–LGR4 interaction in the context of inflammation (Table 1).

### Macrophage modulation

As previously mentioned, the LGR4-Gαi1/3-Gab1-Erk cascade was shown to be activated by RSPO3 [68]. One of the signaling molecules in this pathway, Gαi1/3, was shown to participate in inflammation in a few aspects. Namely, it was shown that Gαi1/3 regulates macrophage polarization, which was associated with increased NF-κB production [74]. Furthermore, Gαi1/3 mediates LPS-induced TLR4 activation, as well as TLR4 endocytosis in macrophages, at the endosome level. Therefore, the LPS-induced cascade was proposed to be mediated by Gαi1/3-Gab1 interaction [74]. Based on these considerations, the question arises is this inflammatory pathway, speaking in favor of M1 type macrophages (Gαi1/3-Gab1), at least in part mediated by RSPO3 and LGR4, since it has been shown that Gαi1/3 was found in their (RSPO3-LGR4) downstream cascade. LGR4 is already known as a regulator of macrophage-related immunological processes. In the experimental model of myocardial infarction, it has been shown that LGR4 potentiates inflammatory modulation of infarct macrophages through AP (activator protein)-1-CREB pathway activation [73]. Targeting LGR4 macrophages might be suitable for early therapeutic interventions to attenuate myocardial injury and enhance wound healing following acute myocardial infarction.

### Respiratory system - lungs

An in vitro study on non-small cell lung cancer (NSCLC) cells demonstrated that RSPO3 overexpression enhances radiosensitivity by inducing pyroptosis. This form of programmed cell death was mediated through activation of the NLRP3 inflammasome and the β-catenin/NF-κB signaling cascade [75]. Once activated, NLRP3 inflammasome stimulates secretion of IL-1ß and IL-18 [76]. RSPO3-driven anti-inflammatory action was shown in the lungs, as well. Namely, RSPO3 was found to be secreted from the lung endothelial cells in response to the inflammatory injury and thereafter activates the Wnt-β-catenin pathway in interstitial macrophages. Once this cascade is activated, it further stimulates the production of α-ketoglutarate, which serves as a cofactor for ten-eleven translocation 2 (TET2), leading to DNA hydroxymethylation [70]. Based on these findings, the authors proposed metabolic-epigenetic events as the mechanism of RSPO3 anti-inflammatory actions. Furthermore, it has been shown that RSPO3 secreted from the lung endothelial cells exerts regenerative potential mediated by its binding to LGR4 receptor, leading to subsequent activation of β-catenin and integrin-linked kinase (ILK)/serine/threonine protein kinase Akt (AKT) downstream cascade [71].

### Gastrointestinal system- stomach and large intestine

Contributing further to the oncogenic potential of the RSPO3, it was suggested that RSPO3-LGR4 interaction mediates events upon Helicobacter pylori (H. pylori) infection, which is known to be a major risk factor for the development of gastric cancer [77,78]. Moreover, it was reported that RSPO3 increases LGR5^−^/AXIN2^+^ gastric stem cells proliferation and expansion, suggesting that this action is mediated by LGR4 [79]. Furthermore, stromal RSPO3 overexpression has been shown in H. pylori infection, leading to the proliferation of AXIN2^+^ stem cells [79]. Another experimental study in mice showed enhanced oxyntic glandular proliferation following H. pylori infection, as part of long-term regeneration induced by RSPO3. Premalignant metaplastic changes followed this glandular proliferation, which was a response to chronic H. Pylori infection. According to this study, RSPO3 drives adaptive responses to chronic infection in the form of glandular hyperplasia through the RSPO3-YAP (yes-associated protein) cascade. Transcriptome data from this research protocol suggest that mTOR signaling and IL-33 contribute to the YAP response to chronic H. Pylori infection [80]. In line with these results, another study in mice reported YAP as a mediator of epithelial regenerative capacity after DSS-induced colitis [81]. Furthermore, gastric stem cells’ proliferation in response to H. Pylori infection was driven by LGR4-NF-kB interaction activated by RSPO3. In addition, results from this study showed that LGR4 regulates the LGR5 expression pattern, and that both receptors are needed for H. pylori-related inflammation and glandular proliferation induced by RSPO3, indicating LGR5 itself is not sufficient for these processes [82]. Apart from its LGR4-mediated effects, RSPO3 has been shown to activate LGR5^+^ gastric stem cells to undergo proliferation and secretion of intelectin-1, providing evidence of its antimicrobial action [83]. Despite new insights into the RSPO3-LGR4 interaction related to H. pylori infection, more studies are needed to further investigate their interplay in more detail.

RSPO3 showed beneficial effects on necrotizing enterocolitis (NEC) mediated by the downstream regulation of adenosine 5’-monophosphate-activated protein kinase α (AMPKα). This study also revealed that amniotic fluid stem cells (AFSCs) exert protective effects on NEC progression through activation of the RSPO3/AMPKα signaling axis, indicating AFSCs application for NEC therapy in the clinical setting [84].

LGR4 is well known for its important role in maintaining intestinal stem cells’ function in both physiological and pathological conditions. For example, it has been found that LGR4 exerts protective effects from DSS-induced colitis in the mouse experimental model. This study reported improved experimental colitis in LGR4 mutant mice through inhibition of glycogen synthase kinase 3 beta (GSK-3β), indicating an important role of LGR4 in inflammatory bowel disease (IBD) recovery [72]. In the context of intestinal inflammation, it is worth mentioning that during the active phase of inflammation, interferon-gamma (INF-γ) is secreted and further, through phosphoinositide 3- kinase (PI3K)/AKT signaling, activates β-catenin, promoting epithelial proliferation [85]. Additionally, it was reported that PI3K-AKT signaling, in addition to Wnt pathway, promotes β-catenin production during intestinal inflammation, promoting progenitor cell activation and progression from chronic colitis to colitis-associated cancer [86]. Findings from this study suggest PI3K, AKT, and β-catenin as biomarkers of dysplasia onset in the colon epithelium. Having in mind PI3K and AKT activation in intestinal inflammation, the question arises whether this cascade is activated in inflammatory conditions, at least in part by RSPO3-LGR4 interaction.

### Vascular system- endothelial cells

Endothelial cell dysfunction in chronic inflammatory processes is one of the pathoanatomic substrates for increased vascular permeability. One *in vitro* study on human primary vascular endothelial cells showed that RSPO3, in synergy with pro-inflammatory mediator IL-1, disrupts the endothelial barrier integrity [87]. Whether RSPO3 cooperates with IL-1 in contributing to endothelial dysfunction under chronic inflammatory conditions remains elusive. Arterial occlusion was performed in mice to develop an ischemic brain model in a study conducted by Shimamura et al. [88]. Results of this study showed RSPO3 and LGR4 expressions in microglia, neural cells, and endothelial cells. Furthermore, the RSPO3-LGR4 interaction has been found to decrease TLR4, TLR3, and TLR9-induced inflammatory response by reducing the expression of related cytokines [88]. Considering the results of these experiments, enhancing the RSPO3-LGR4 signaling might find clinical application in the treatment of ischemic stroke. Namely, by modulating the production of inflammatory cytokines via the RSPO3–LGR4 signaling axis, protective effects on neuronal cells might be anticipated.

### Network Analysis of RSPO3-LGR4 Signaling related to Inflammation

**Figure 2:**
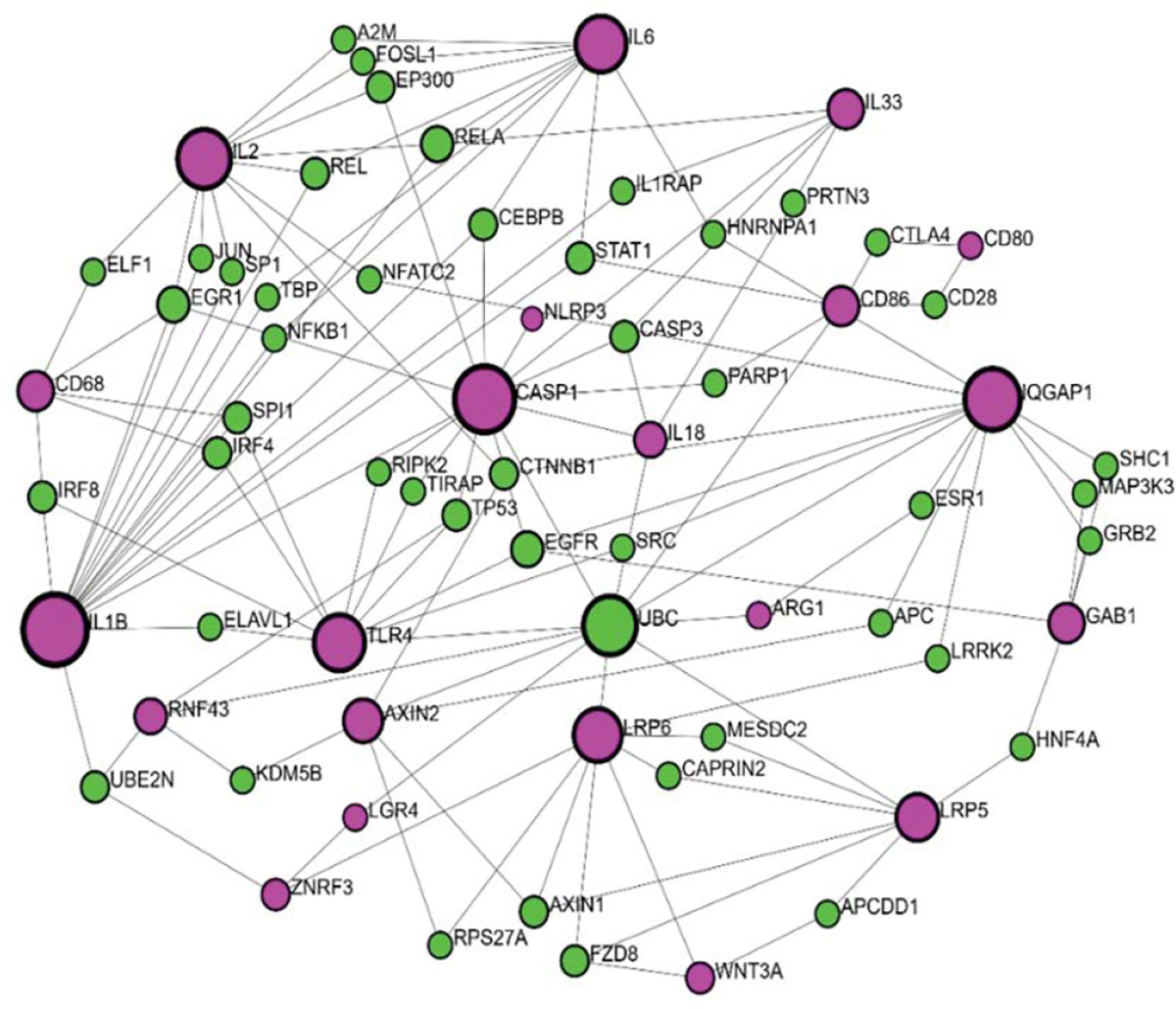
IMEx interactome network analysis for the–RSPO3-LGR4 protein-protein interactions (PPI). Rose circles (input proteins), green circles (output proteins).

According to all the above-mentioned, it can be said that RSPO3-LGR4 interactions related to inflammatory processes are at certain points identified but not completely clarified. In order to get better insight into RSPO3-LGR4 interactions regarding this matter, we performed network analysis using Network analyst (www.networkanalyst.ca). To analyse interactions of RSPO3 and LGR4 with other proteins, we used a tool called Protein-protein interactions (PPI). A gene input list was created based on a literature review of molecules identified to participate in RSPO3 and LGR4 signaling as upstream or downstream regulators, inflammatory mediators, or linking molecules. Gene input list included core Wnt pathway proteins (RSPO3, LGR4, ZNRF3, RNF43, WNT2, WNT3A, FZD, LRP5, LRP6); coresponding downstream molecules (CTNNB1 (β-catenin), AXIN2, MAPK, ERK, GAB1); interconecting molecule (IQGAP1); inflammatory mediators related to RSPO3 and LGR4 signalization (NF-κB, IL-1β, IL1, NLRP3, TLR4, CREB, IL33, PI3K) as well as downstream cytokines (Caspase-1, IL18, TNF-α, IL2, IL6, IL8). Since LGR4 modulates macrophage function in inflammation, macrophage markers were also included in the gene input list (CD68, CD80, CD86, CD163, Arginase-1). What first came to our knowledge from the network analysis using IMEx interactome is that there were no direct interactions between RSPO3 and LGR4 (Figure 2). Furthermore, the RSPO3 gene wasn’t present in this setting. LGR4 showed direct interactions with UBC and ZNRF3. In addition, UBC showed a high level of interaction with other molecules, related to the inflammatory cascade, such as CD86, IL18, CASP1, TLR4, and ARG1, suggesting its role in the inflammation and making it a candidate for further transcription factor (TF)-gene interaction analysis.

### Transcription Factors in the Regulation of RSPO3 and LGR4 Interactions

**Figure 3:**
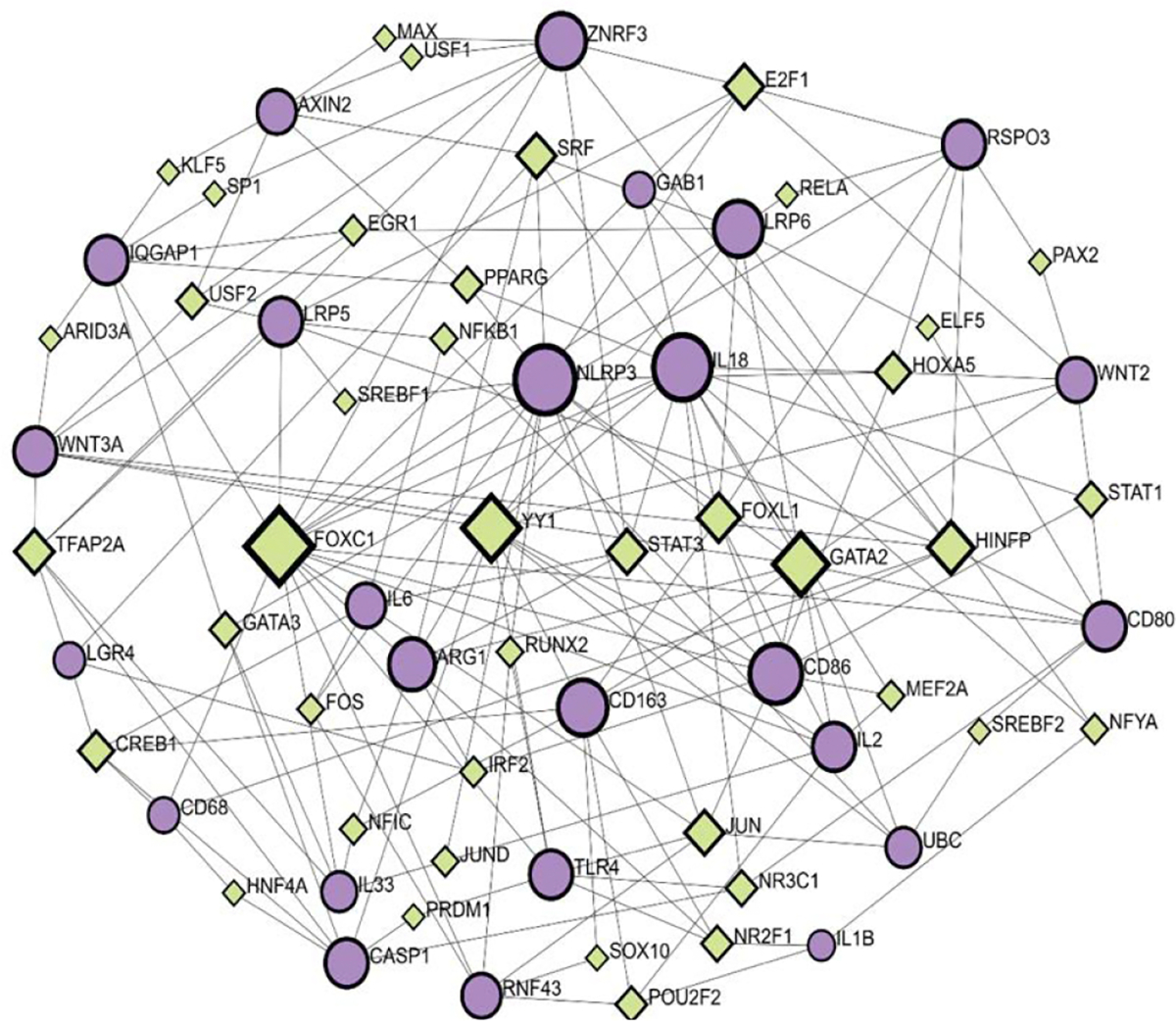
Transcription factor-gene interactions based on RSOP3-LGR4 signaling in inflammation using the JASPAR database. Purple circles (input genes); green squares (output transcription factors).

Network analysis using the Gene-Regulatory Networks (GRN) tool revealed interactions between genes included in RSPO3-LGR4 pathways in the context of inflammation and output TF, using the JASPAR database (Figure 3). Gene input list included the same genes as used for PPI, in addition to UBC (RSPO3, LGR4, ZNRF3, RNF43, WNT2, WNT3A, FZD, LRP5, LRP6, CTNNB1 (β-catenin), AXIN2, MAPK, ERK, GAB1, IQGAP1, NF-κB, IL-1β, IL1, NLRP3, TLR4, CREB, IL33, PI3K, Caspase-1, IL18, TNF-α, IL2, IL6, IL8, CD68, CD80, CD86, CD163, Arginase-1, UBC).

Predictions presented by TF-gene regulatory network indicate that RSPO3 regulation includes the following TF: E2F1, PAX2, HOXA5, RELA, and HINFP by regulating the following gene expression: GAB1, LRP6, CD86, WNT2, CD80, and CD86 with a betweenness of 46.1; 1.15; 23.85; 2.39 and 100.99, respectively. Distant from RSPO3 node, LGR4 interaction was presented in the network. Furthermore, LGR4 was found to communicate to CD68, WNT3A, CD86, AXIN2 and GAB1, regulated by the following TF: CREB1, TFAP2A, IRF2 and SRF with a betweenness values of 37.39; 61.84; 9.61 and 87.06. The highest betweenness value of 487.42 was shown for FOXC1, indicating its strong influence in the regulatory network. FOXC1 was predicted to interact with RSPO3 via CD80 and WNT2, transcriptionally regulated by STAT1 (betweenness 12.96) and PAX2 (betweenness 1.15). FOXC1 interaction with LGR4 was predicted as well, through the cascade of genes (LRP5, AXIN2, IQGAP1, WNT3A) regulated by TF as follows: USF2, KLF5, ARID3A, TFAP2A.

In addition, RSPO3-LGR4 interaction was predicted to be mediated by the following genes in the network: ZNRF3, AXIN2, IQGAP1, WNT3A, and regulated by TF such as E2F1, MAX, USF1, KLF5, ARID3A, and TFAP2A, with the betweenness as follows: 46.11; 3.42; 3.42; 3.91; 2.67 and 61.84, respectively. The highest betweenness in this cascade belongs to TFAP2A, which was further predicted to directly interact with inflammatory mediators such as Caspase 1 and IL33. RSPO3 and LGR4 were predicted to interact in a cascade made of additional genes like CD80, TLR4, CASP1, and CD68, regulated by HINFP, NRC31, PRDM1, HNF4A, and CREB1, respectively.

According to the presented *in silico* model of RSPO3-LGR4 inflammatory-related interactions, notable predictions between input genes and output TF were identified and proposed. What is still considered unknown in this context is the nature of these interactions, in terms of upstream or downstream regulation. In order to investigate it in more detail, some of the *in silico* identified pathways should be selected for further analysis and verification in an *in vitro* model.

### MicroRNAs in the Regulation of RSPO3 and LGR4 Signaling

MicroRNAs (miRNAs) represent short, non-coding RNAs that play an important role in gene expression at the post-transcriptional level, after the transcription of the gene to mRNA occurs [89]. They bind to the target mRNA, resulting in either mRNA degradation or the prevention of its translation to a protein [90]. A growing body of evidence lists miRNA regulation of inflammatory cascades, indicating their altered expression patterns mainly in immune cells [91]. Different stages of the inflammation process (initial phase, progression, resolution) are regulated by miRNAs through both positive and negative feedback loops [92]. In addition, it has been reported that LGR4 signaling is partially regulated by miRNAs.

Therefore, miR-193a-3p was shown to induce an inflammatory response by targeting LGR4 and subsequent production of IL-1β, IL-6, and TNF-α, as presented in an experimental model of endometritis [93]. Another experimental endometritis model also reports LGR4 as a miRNA target. In this study, miR-34a was shown to be upregulated by IL-1β, leading to LGR4 downregulation and subsequent inflammation [94]. The miR-34 family also plays a role in wound inflammation, primarily through the miR-34–LGR4 axis. This interaction impairs the inflammatory response of keratinocytes, ultimately resulting in delayed wound closure and the persistence of chronic venous ulcers [95]. Furthermore, LGR4 was shown to be the target of miR-361–5p in a sepsis-induced cardiac impairment model [96].

**Figure 4:**
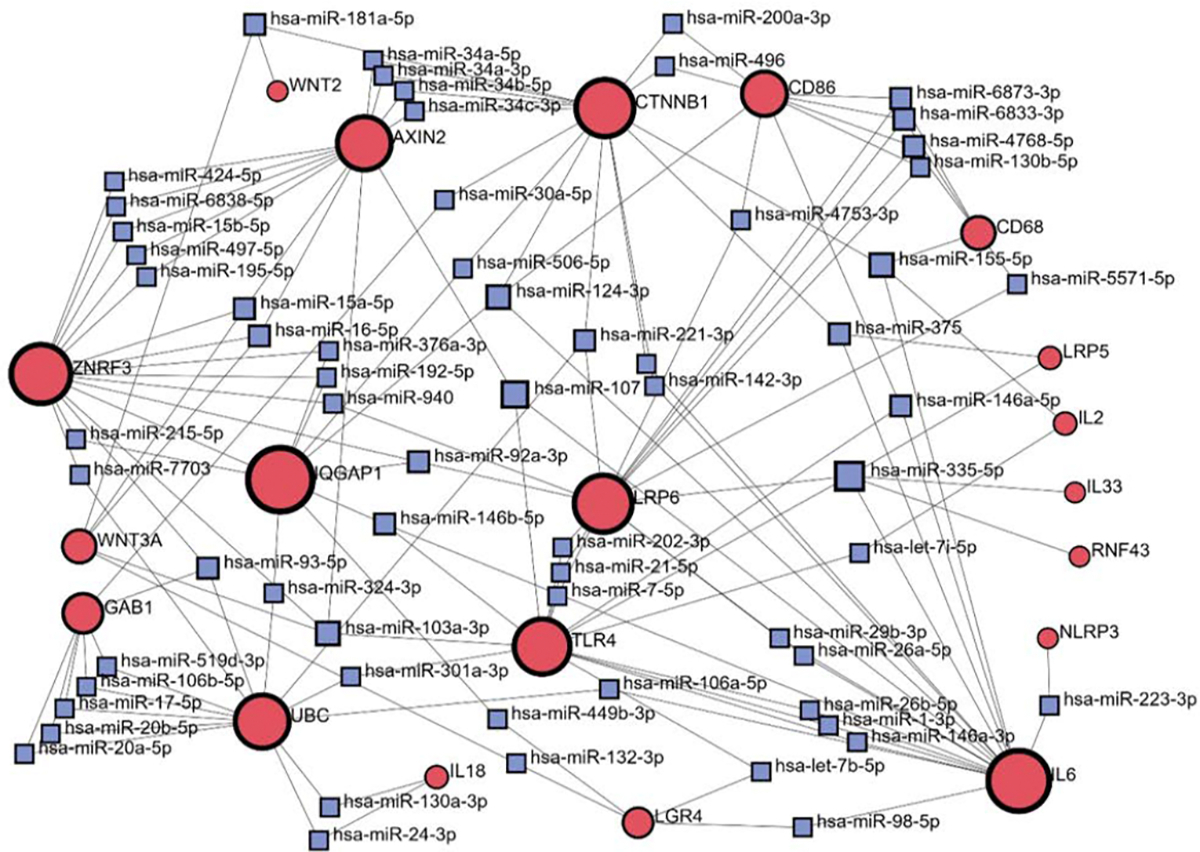
Network analysis for the microRNAs-gene interactions using miRTarBase v9.0. Red circles (input genes), blue squares (output microRNAs).

To explore in more detail which miRNAs participate in the regulation of RSPO3-LGR4 signaling related to inflammation, we provided network analysis using the miRNA-gene interaction database, miRTarBase v9.0 (Figure 4). Input gene list included previously mentioned genes, which are part of the core Wnt signaling pathway, its downstream effectors, or inflammatory mediators. Based on the network analysis, predicted interactions between CTNNB1 and AXIN2 has been suggested to be regulated by miRNAs-34 family (has-mir-34a-5p; has-mir-34a-3p; has-mir-34b-5p; has-mir-34c-3p). To communicate with CD86, CTNNB1 showed predicted regulation by has-mir-200a-3p and has-mir-496. In the context of LGR4 interactions, different miRNAs (has-mir-98–5b; has-let-7b-5p; has-mir-449–3p) were predicted to regulate the LGR4 signaling including following genes (IL6; TLR4; IQGAP1). Notable output from this analysis is the absence of the RSPO3 node in the miRNAs-gene network. This finding could be interpreted in line with the literature data, that most of the miRNA regulations are referred to the LGR4 molecule. These findings suggest a potential translational application of specific microRNAs by targeting LGR4 to modulate the RSPO3–LGR4 signaling pathway related to inflammation. Taken all together, miRNA knockdown or other modulation may represent a promising therapeutic strategy for the treatment of inflammatory conditions.

## Conclusion

According to the RSPO3 and LGR4 signaling pathways reported in the literature, they participate in numerous physiological processes of tissue development, as well as in pathophysiological mechanisms of disease and oncogenesis in various organs. Significant expressions of RSPO3 and LGR4 have been observed in the gastrointestinal tract, bones, or endothelial cells of the brain and lungs. RSPO3 and LGR4 interactions in inflammation, as documented in various experimental models, were taken into consideration during the planning of our *in silico* analysis. The network analysis aimed to explain in more detail the mechanisms of regulation of RSPO3-LGR4 signaling, taking into account other constituents of the Wnt cascade as well as related inflammatory mediators. The results of this *in silico* analysis revealed some new regulatory mechanisms in terms of TF and miRNAs in relation to RSPO3 and LGR4 cascade in inflammation. It is of special importance to emphasize that some of the regulatory molecules are potential targets for therapeutic interventions in order to establish better control over inflammatory processes. Extracellular domain of LGR4 is one of them. Nevertheless, further experimental studies are needed to examine predicted interactions revealed in the network analysis. For better insight, a tissue/cell-specific approach is preferable.

## Figures and Tables

**Table 1:** Tissue and Cell-based overview of RSPO3 and LGR4 signaling in inflammation.

Tissue/cell type	Regulatory pathway	Key features/effects	Study type	Reference
**Macrophages**	AP1-CREB activation	LGR4 potentiates inflammatory modulation	Experimental model of myocardial infarction	[73]
**Lungs**	Activation of NLRP3 and β-catenin-NF-kB	RSPO3-mediated pyroptosis	In vitro model of lung cancer cells	[75]
	Activation of the Wnt-β-catenin pathway	Anti-inflammatory action of endothelial-derived RSPO3	In vitro model on the lung interstitial macrophages	[70]
	RSPO3-LGR4-ILK-AKT	Regenerative potential of endothelial-derived RSPO3	In vitro model of inflammatory vascular injury	[71]
**Stomach/Intestine**	RSPO3-YAP cascade activation; mTOR signaling and IL-33 contribute to the YAP response	Response to chronic H. pylori infection in the form of glandular hyperplasia	Experimental study in mice; Transcriptome data	[80]
	RSPO3-LGR4-NF-kB	Response to H. pylori infection in the form of gastric stem cells’ proliferation	Conditional knockout mice model	[82]
	RSPO3/AMPKα	RSPO3 beneficial effects on necrotizing enterocolitis	In vitro cell model induced by LPS	[84]
	Inhibition of glycogen synthase kinase 3 beta (GSK-3β)	LGR4 beneficial effects on inflammatory bowel disease (IBD)	Mouse experimental model of DSS-induced colitis	[72]
	PI3K-AKT	Promoting progenitor cell activation and progression from chronic colitis to colitis-associated cancer	Mouse experimental model of colitis	[86]
**Endothelial cells**	RSPO3 in synergy with pro-inflammatory mediator IL-1	Disruption of the endothelial barrier integrity	In vitro study on human primary vascular endothelial cells	[87]
	RSPO3-LGR4	Decrease of TLR4, TLR3, and TLR9-induced inflammatory response	Ischemic brain model in mice	[88]

## References

[1] Yoon JK, Lee JS. Cellular signaling and biological functions of R-spondins. Cellular Signalling 24 (2012): 369–377. 21982879 10.1016/j.cellsig.2011.09.023 PMC3237830

[2] Chen JZ, Wang S, Tang R, Cloning and identification of a cDNA that encodes a novel human protein with thrombospondin type I repeat domain, hPWTSR. Molecular Biology Reports 29 (2002): 287–292. 12463421 10.1023/a:1020479301379

[3] Kazanskaya O, Glinka A, del Barco Barrantes I, R-Spondin2 is a secreted activator of Wnt/β-catenin signaling and is required for Xenopus myogenesis. Developmental Cell 7 (2004): 525–534. 15469841 10.1016/j.devcel.2004.07.019

[4] Zebisch M, Xu Y, Krastev C, Structural and molecular basis of ZNRF3/RNF43 transmembrane ubiquitin ligase inhibition by the Wnt agonist R-spondin. Nature Communications 4 (2013): 2787. 10.1038/ncomms3787 PMC3905715 24225776

[5] Lebensohn AM, Rohatgi R. R-spondins can potentiate WNT signaling without LGRs. Elife 7 (2018): e33126. 29405118 10.7554/eLife.33126 PMC5800842

[6] Wang D, Huang B, Zhang S, Structural basis for R-spondin recognition by LGR4/5/6 receptors. Genes & Development 27 (2013): 1339–1344. 10.1101/gad.219360.113 PMC3701189 23756652

[7] Kazanskaya O, Ohkawara B, Heroult M, The Wnt signaling regulator R-spondin 3 promotes angioblast and vascular development. Development 135 (2008): 3655–3664. 18842812 10.1242/dev.027284

[8] Sugimoto A, Saito Y, Wang G, Hepatic stellate cells control liver zonation, size and functions via R-spondin 3. Nature 625 (2025): 1–10. 10.1038/s41586-025-08677-w PMC12003176 40074890

[9] Takahashi T, Li Y, Chen W, RSPO3 is a novel contraction-inducible factor identified in an in vitro exercise model using primary human myotubes. Scientific Reports 12 (2022): 14291. 35995979 10.1038/s41598-022-18190-z PMC9395423

[10] Nagano K, Yamana K, Saito H, R-spondin 3 deletion induces Erk phosphorylation to enhance Wnt signaling and promote bone formation in the appendicular skeleton. Elife 11 (2022): e84171. 36321691 10.7554/eLife.84171 PMC9681208

[11] Cambier L, Plate M, Sucov HM, Nkx2–5 regulates cardiac growth through modulation of Wnt signaling by R-spondin3. Development 141 (2014): 2959–2971. 25053429 10.1242/dev.103416 PMC4197672

[12] Da Silva F, Massa F, Motamedi FJ, Myocardial-specific R-spondin3 drives proliferation of the coronary stems primarily through the Leucine Rich Repeat G Protein coupled receptor LGR4. Developmental Biology 441 (2018): 42–51. 29859889 10.1016/j.ydbio.2018.05.024 PMC6365680

[13] Harnack C, Berger H, Antanaviciute A, R-spondin 3 promotes stem cell recovery and epithelial regeneration in the colon. Nature Communications 10 (2019): 4368. 10.1038/s41467-019-12349-5 PMC6761174 31554819

[14] Rocha AS, Vidal V, Mertz M, The angiocrine factor Rspondin3 is a key determinant of liver zonation. Cell Reports 13 (2015): 1757–1764. 26655896 10.1016/j.celrep.2015.10.049

[15] Nilsson KH, Henning P, El Shahawy M, RSPO3 is important for trabecular bone and fracture risk in mice and humans. Nature Communications 12 (2021): 4923. 10.1038/s41467-021-25124-2 PMC8363747 34389713

[16] Shi GX, Mao WW, Zheng XF, The role of R-spondins and their receptors in bone metabolism. Progress in Biophysics and Molecular Biology 122 (2016): 93–100. 27237581 10.1016/j.pbiomolbio.2016.05.012

[17] Ter Steege EJ, Bakker ER. The role of R-spondin proteins in cancer biology. Oncogene 40 (2021): 6469–6478. 34663878 10.1038/s41388-021-02059-y PMC8616751

[18] Kato S, Matsubara M, Matsuo T, Leucine-rich repeat-containing G protein-coupled receptor-4 (LGR4, Gpr48) is essential for renal development in mice. Nephron Experimental Nephrology 104 (2006): e63–e75. 16785743 10.1159/000093999

[19] Garcia MI. LGRs receptors as peculiar GPCRs involved in cancer. Journal of Stem Cell Research and Medicine 2 (2017): 6–9.

[20] Yi J, Xiong W, Gong X, Analysis of LGR4 receptor distribution in human and mouse tissues. PLoS One 8 (2013): e78144. 24205130 10.1371/journal.pone.0078144 PMC3804454

[21] Mustata RC, Van Loy T, Lefort A, Lgr4 is required for Paneth cell differentiation and maintenance of intestinal stem cells ex vivo. EMBO Reports 12 (2011): 558–564. 21508962 10.1038/embor.2011.52 PMC3128273

[22] Zhang N, Yuan M, Wang J. LGR4: a new receptor member in endocrine and metabolic diseases. Endocrine Reviews 44 (2023): 647–667. 36791020 10.1210/endrev/bnad003 PMC10335173

[23] Kinzel B, Pikiolek M, Orsini V, Functional roles of Lgr4 and Lgr5 in embryonic gut, kidney and skin development in mice. Developmental Biology 390 (2014): 181–190. 24680895 10.1016/j.ydbio.2014.03.009

[24] Glinka A, Dolde C, Kirsch N, LGR4 and LGR5 are R-spondin receptors mediating Wnt/β-catenin and Wnt/PCP signalling. EMBO Reports 12 (2011): 1055–1061. 21909076 10.1038/embor.2011.175 PMC3185347

[25] Carmon KS, Gong X, Lin Q, R-spondins function as ligands of the orphan receptors LGR4 and LGR5 to regulate Wnt/β-catenin signaling. Proceedings of the National Academy of Sciences 108 (2011): 11452–11457. 10.1073/pnas.1106083108 PMC3136304 21693646

[26] Luo J, Zhou W, Zhou X, Regulation of bone formation and remodeling by G-protein-coupled receptor 48. Molecular and Cellular Biology 29 (2009): 3435–3445. 19605502 10.1242/dev.033571 PMC2730404

[27] Mancini A, Howard SR, Marelli F, LGR4 deficiency results in delayed puberty through impaired Wnt/β-catenin signaling. JCI Insight 5 (2020): e133434. 32493844 10.1172/jci.insight.133434 PMC7308048

[28] Li Z, Zhang W, Mulholland MW. LGR4 and its role in intestinal protection and energy metabolism. Frontiers in Endocrinology 6 (2015): 131. 26379625 10.3389/fendo.2015.00131 PMC4548225

[29] Li JY, Chai B, Zhang W, LGR4 and its ligands, R-spondin 1 and R-spondin 3, regulate food intake in the hypothalamus of male rats. Endocrinology 155 (2014): 429–440. 24280058 10.1210/en.2013-1550 PMC3891940

[30] Yue Z, Yuan Z, Zeng L, LGR4 modulates breast cancer initiation, metastasis, and cancer stem cells. FASEB Journal 32 (2018): 2422–2433. 29269400 10.1096/fj.201700897R PMC5901378

[31] Zeng Z, Ji N, Yi J, LGR4 overexpression is associated with clinical parameters and poor prognosis of serous ovarian cancer. Cancer Biomarkers 28 (2020): 65–72. 32176632 10.3233/CBM-191145 PMC12662327

[32] Thankam FG, Boosani CS, Dilisio MF, Epigenetic mechanisms and implications in tendon inflammation. International Journal of Molecular Medicine 43 (2019): 3–14. 30387824 10.3892/ijmm.2018.3961 PMC6257858

[33] Nguyen AH, Berim IG, Agrawal DK. Chronic inflammation and cancer: emerging roles of triggering receptors expressed on myeloid cells. Expert Review of Clinical Immunology 11 (2015): 849–857. 25954917 10.1586/1744666X.2015.1043893 PMC4829915

[34] Truong R, Thankam FG, Agrawal DK. Immunological mechanisms underlying sterile inflammation in the pathogenesis of atherosclerosis: potential sites for intervention. Expert Review of Clinical Immunology 17 (2021): 37–50. 33280442 10.1080/1744666X.2020.1860757 PMC7906938

[35] Nandipati KC, Subramanian S, Agrawal DK. Protein kinases: mechanisms and downstream targets in inflammation-mediated obesity and insulin resistance. Molecular and Cellular Biochemistry 426 (2017): 27–45. 27868170 10.1007/s11010-016-2878-8 PMC5291752

[36] Hall SC, Agrawal DK. Toll-like receptors, triggering receptor expressed on myeloid cells family members and receptor for advanced glycation end-products in allergic airway inflammation. Expert Review of Respiratory Medicine 10 (2016): 171–184. 26678062 10.1586/17476348.2016.1133303 PMC4955846

[37] Biben C, Harvey RP. Homeodomain factor Nkx2–5 controls left/right asymmetric expression of bHLH gene eHand during murine heart development. Genes & Development 11 (1997): 1357–1369. 9192865 10.1101/gad.11.11.1357

[38] Tan C, Norden PR, Yu W, Endothelial FOXC1 and FOXC2 promote intestinal regeneration after ischemia-reperfusion injury. EMBO Reports 24 (2023): e56030. 37154714 10.15252/embr.202256030 PMC10328078

[39] Ter Steege EJ, Bakker ERM. The role of R-spondin proteins in cancer biology. Oncogene 40 (2021): 6469–6478. 34663878 10.1038/s41388-021-02059-y PMC8616751

[40] Storm EE, Durinck S, de Sousa e Melo F, Targeting PTPRK-RSPO3 colon tumours promotes differentiation and loss of stem-cell function. Nature 529 (2016): 97–100. 26700806 10.1038/nature16466

[41] Wu G, Wang D, Xiong F, Upregulation of RSPO3 via targeted promoter DNA demethylation inhibits the progression of cholangiocarcinoma. Clinical Epigenetics 15 (2023): 177. 37932819 10.1186/s13148-023-01592-9 PMC10629118

[42] Nilsson KH, Wu J, Gustafsson KL, Estradiol and RSPO3 regulate vertebral trabecular bone mass independent of each other. American Journal of Physiology-Endocrinology and Metabolism 322 (2022): E211–E218. 35068191 10.1152/ajpendo.00383.2021 PMC8896994

[43] Nusse R, Varmus HE. Many tumors induced by the mouse mammary tumor virus contain a provirus integrated in the same region of the host genome. Cell 31 (1982): 99–109. 6297757 10.1016/0092-8674(82)90409-3

[44] Rashdan NA, Sim AM, Cui L, Osteocalcin regulates arterial calcification via altered Wnt signaling and glucose metabolism. Journal of Bone and Mineral Research 35 (2020): 357–367. 31596966 10.1002/jbmr.3888

[45] Wu W, Xiao Z, Chen Y, CD39 produced from human GMSCs regulates the balance of osteoclasts and osteoblasts through the Wnt/β-Catenin pathway in osteoporosis. Molecular Therapy 28 (2020): 1518–1532. 32304668 10.1016/j.ymthe.2020.04.003 PMC7264439

[46] Singh S, Mishra A, Mohanbhai SJ, Axin-2 knockdown promotes mitochondrial biogenesis and dopaminergic neurogenesis by regulating Wnt/β-catenin signaling in a rat model of Parkinson’s disease. Free Radical Biology & Medicine 129 (2018): 73–87. 30176346 10.1016/j.freeradbiomed.2018.08.033

[47] Taniguchi K, Roberts LR, Aderca IN, Mutational spectrum of beta-catenin, AXIN1, and AXIN2 in hepatocellular carcinomas and hepatoblastomas. Oncogene 21 (2002): 4863–4871. 12101426 10.1038/sj.onc.1205591

[48] Nusse R, Clevers H. Wnt/β-catenin signaling, disease, and emerging therapeutic modalities. Cell 169 (2017): 985–999. 28575679 10.1016/j.cell.2017.05.016

[49] Hayat R, Manzoor M, Hussain A. Wnt signaling pathway: a comprehensive review. Cell Biology International 46 (2022): 863–877. 35297539 10.1002/cbin.11797

[50] Prunier C, Hocevar BA, Howe PH. Wnt signaling: physiology and pathology. Growth Factors 22 (2004): 141–150. 15518237 10.1080/08977190410001720860

[51] MacDonald BT, He X. Frizzled and LRP5/6 receptors for Wnt/β-catenin signaling. Cold Spring Harbor Perspectives in Biology 4 (2012): a007880. 23209147 10.1101/cshperspect.a007880 PMC3504444

[52] Logan CY, Nusse R. The Wnt signaling pathway in development and disease. Annual Review of Cell and Developmental Biology 20 (2004): 781–810. 10.1146/annurev.cellbio.20.010403.113126 15473860

[53] Liu J, Xiao Q, Xiao J, Wnt/β-catenin signalling: function, biological mechanisms, and therapeutic opportunities. Signal Transduction and Targeted Therapy 7 (2022): 3. 34980884 10.1038/s41392-021-00762-6 PMC8724284

[54] Reyes M, Flores T, Betancur D, Wnt/β-catenin signaling in oral carcinogenesis. International Journal of Molecular Sciences 21 (2020): 13. 10.3390/ijms21134682 PMC7369957 32630122

[55] Brannon M, Brown JD, Bates R, XCtBP is a XTcf-3 co-repressor with roles throughout Xenopus development. Development 126 (1999): 3159–3170. 10375506 10.1242/dev.126.14.3159

[56] Duan P, Bonewald LF. The role of the Wnt/β-catenin signaling pathway in formation and maintenance of bone and teeth. International Journal of Biochemistry & Cell Biology 77 (2016): 23–29. 27210503 10.1016/j.biocel.2016.05.015 PMC4958569

[57] Shen Y, Wang J, Dai Y, RSPO3 promotes proliferation and self-renewal of limbal epithelial stem cells through a WNT/β-catenin-independent signaling pathway. Investigative Ophthalmology & Visual Science 66 (2025): 8. 10.1167/iovs.66.1.8 PMC11717127 39760688

[58] Lee H, Seidl C, Sun R, R-spondins are BMP receptor antagonists in Xenopus early embryonic development. Nature Communications 11 (2020): 5570. 10.1038/s41467-020-19373-w PMC7642414 33149137

[59] Seifert JR, Mlodzik M. Frizzled/PCP signalling: a conserved mechanism regulating cell polarity and directed motility. Nature Reviews Genetics 8 (2007): 126–138. 10.1038/nrg2042 17230199

[60] De A Wnt/Ca2+ signaling pathway: a brief overview. Acta Biochimica et Biophysica Sinica 43 (2011): 745–756. 21903638 10.1093/abbs/gmr079

[61] Scholz B, Korn C, Wojtarowicz J, Endothelial RSPO3 controls vascular stability and pruning through non-canonical WNT/Ca2+/NFAT signaling. Developmental Cell 36 (2016): 79–93. 26766444 10.1016/j.devcel.2015.12.015

[62] Hao HX, Xie Y, Zhang Y, ZNRF3 promotes Wnt receptor turnover in an R-spondin-sensitive manner. Nature 485 (2012): 195–200. 22575959 10.1038/nature11019

[63] Moad HE, Pioszak AA. Reconstitution of R-spondin:LGR4:ZNRF3 adult stem cell growth factor signaling complexes with recombinant proteins produced in Escherichia coli. Biochemistry 52 (2013): 7295–7304. 24050775 10.1021/bi401090h PMC3836688

[64] Peng WC, de Lau W, Madoori PK, Structures of Wnt-antagonist ZNRF3 and its complex with R-spondin 1 and implications for signaling. PLoS One 8 (2013): e83110. 24349440 10.1371/journal.pone.0083110 PMC3861454

[65] Zhang M, Zhang P, Liu Y, RSPO3-LGR4 regulates osteogenic differentiation of human adipose-derived stem cells via ERK/FGF signalling. Scientific Reports 7 (2017): 42841. 28220828 10.1038/srep42841 PMC5318871

[66] White CD, Erdemir HH, Sacks DB. IQGAP1 and its binding proteins control diverse biological functions. Cellular Signalling 24 (2012): 826–834. 22182509 10.1016/j.cellsig.2011.12.005 PMC3268868

[67] Carmon KS, Gong X, Yi J, RSPO-LGR4 functions via IQGAP1 to potentiate Wnt signaling. Proceedings of the National Academy of Sciences 111 (2014): E1221–E1229. 10.1073/pnas.1323106111 PMC3977305 24639526

[68] Liu TT, Shi X, Hu HW, Endothelial cell-derived RSPO3 activates Gαi1/3-Erk signaling and protects neurons from ischemia/reperfusion injury. Cell Death & Disease 14 (2023): 654. 37805583 10.1038/s41419-023-06176-2 PMC10560285

[69] Luo J, Yang Z, Ma Y, LGR4 is a receptor for RANKL and negatively regulates osteoclast differentiation and bone resorption. Nature Medicine 22 (2016): 539–546. 10.1038/nm.4076 27064449

[70] Zhou B, Magana L, Hong Z, The angiocrine Rspondin3 instructs interstitial macrophage transition via metabolic-epigenetic reprogramming and resolves inflammatory injury. Nature Immunology 21 (2020): 1430–1443. 32839607 10.1038/s41590-020-0764-8 PMC7815054

[71] Zhang H, Liu D, Xu QF, Endothelial RSPO3 mediates pulmonary endothelial regeneration by LGR4-dependent activation of β-catenin and ILK signaling pathways after inflammatory vascular injury. International Journal of Biological Macromolecules 269 (2024): 131805. 38677673 10.1016/j.ijbiomac.2024.131805

[72] Liu S, Qian Y, Li L, Lgr4 gene deficiency increases susceptibility and severity of dextran sodium sulfate-induced inflammatory bowel disease in mice. Journal of Biological Chemistry 288 (2013): 8794–8803. 23393138 10.1074/jbc.M112.436204 PMC3610954

[73] Huang CK, Dai D, Xie H, Lgr4 governs a pro-inflammatory program in macrophages to antagonize post-infarction cardiac repair. Circulation Research 127 (2020): 953–973. 32600176 10.1161/CIRCRESAHA.119.315807

[74] Li X, Wang D, Chen Z, Gαi1 and Gαi3 regulate macrophage polarization by forming a complex containing CD14 and Gab1. Proceedings of the National Academy of Sciences 112 (2015): 4731–4736. 10.1073/pnas.1503779112 PMC4403188 25825741

[75] Li H, Zhang J, Yu B, RSPO3 regulates the radioresistance of non-small cell lung cancer cells via NLRP3 inflammasome-mediated pyroptosis. Radiotherapy and Oncology 200 (2024): 110528. 39245068 10.1016/j.radonc.2024.110528

[76] Satish M, Agrawal DK. Atherothrombosis and the NLRP3 inflammasome – endogenous mechanisms of inhibition. Translational Research 215 (2020): 75–85. 31469975 10.1016/j.trsl.2019.08.003 PMC6889001

[77] Fischer AS, Sigal M. The role of Wnt and R-spondin in the stomach during health and disease. Biomedicines 7 (2019): 2. 10.3390/biomedicines7020044 PMC6631168 31248166

[78] Tomasetti C, Vogelstein B. Cancer etiology: variation in cancer risk among tissues can be explained by the number of stem cell divisions. Science 347 (2015): 78–81. 25554788 10.1126/science.1260825 PMC4446723

[79] Sigal M, Logan CY, Kapalczynska M, Stromal R-spondin orchestrates gastric epithelial stem cells and gland homeostasis. Nature 548 (2017): 451–455. 28813421 10.1038/nature23642

[80] Fischer AS, Müllerke S, Arnold A, R-spondin/YAP axis promotes gastric oxyntic gland regeneration and Helicobacter pylori-associated metaplasia in mice. Journal of Clinical Investigation 132 (2022): 21. 10.1172/JCI151363 PMC9621134 36099044

[81] Yui S, Azzolin L, Maimets M, YAP/TAZ-dependent reprogramming of colonic epithelium links ECM remodeling to tissue regeneration. Cell Stem Cell 22 (2018): 35–49.e7. 29249464 10.1016/j.stem.2017.11.001 PMC5766831

[82] Wizenty J, Müllerke S, Kolesnichenko M, Gastric stem cells promote inflammation and gland remodeling in response to Helicobacter pylori via Rspo3-Lgr4 axis. EMBO Journal 41 (2022): e109996. 35767364 10.15252/embj.2021109996 PMC9251867

[83] Sigal M, Reinés MDM, Müllerke S, R-spondin-3 induces secretory, antimicrobial Lgr5(+) cells in the stomach. Nature Cell Biology 21 (2019): 812–823. 31235935 10.1038/s41556-019-0339-9

[84] Ning N, Wang Q, Li J, Amniotic fluid stem cell attenuated necrotizing enterocolitis progression by promoting Rspo3/AMPKα axis. Immunobiology 228 (2023): 152336. 37173190 10.1016/j.imbio.2023.152336

[85] Nava P, Koch S, Laukoetter MG, Interferon-gamma regulates intestinal epithelial homeostasis through converging beta-catenin signaling pathways. Immunity 32 (2010): 392–402. 20303298 10.1016/j.immuni.2010.03.001 PMC2859189

[86] Lee G, Goretsky T, Managlia E, Phosphoinositide 3-kinase signaling mediates beta-catenin activation in intestinal epithelial stem and progenitor cells in colitis. Gastroenterology 139 (2010): 869–881.e9. 20580720 10.1053/j.gastro.2010.05.037 PMC2930080

[87] Skaria T, Bachli E, Schoedon G. RSPO3 impairs barrier function of human vascular endothelial monolayers and synergizes with pro-inflammatory IL-1. Molecular Medicine 24 (2018): 45. 30157748 10.1186/s10020-018-0048-z PMC6116367

[88] Shimamura M, Hayashi H, Ju N, R-spondin 3/LGR4 (Leucine-Rich Repeat-Containing G Protein-Coupled Receptor 4) axis is a novel inflammatory and neurite outgrowth signaling system in the ischemic brain in mice. Stroke 54 (2023): 1606–1615. 37165865 10.1161/STROKEAHA.122.041970

[89] Supra R, Agrawal DK. Mechanobiology of microRNAs in intervertebral disk degeneration. Journal of Spine Research & Surgery 5 (2023): 1–9. 36777190 10.26502/fjsrs0051 PMC9912327

[90] Bartel DP. MicroRNAs: genomics, biogenesis, mechanism, and function. Cell 116 (2004): 281–297. 14744438 10.1016/s0092-8674(04)00045-5

[91] O’Connell RM, Rao DS, Baltimore D. MicroRNA regulation of inflammatory responses. Annual Review of Immunology 30 (2012): 295–312. 10.1146/annurev-immunol-020711-075013 22224773

[92] Medzhitov R, Horng T. Transcriptional control of the inflammatory response. Nature Reviews Immunology 9 (2009): 692–703. 10.1038/nri2634 19859064

[93] Yin B, Umar T, Ma X, MiR-193a-3p targets LGR4 to promote the inflammatory response in endometritis. International Immunopharmacology 98 (2021): 107718. 34139630 10.1016/j.intimp.2021.107718

[94] Ma X, Yin B, Guo S, Enhanced expression of miR-34a enhances Escherichia coli lipopolysaccharide-mediated endometritis by targeting LGR4 to activate the NF-κB pathway. Oxidative Medicine and Cellular Longevity 2021 (2021): 1744754. 34504639 10.1155/2021/1744754 PMC8422159

[95] Wu J, Li X, Li D, MicroRNA-34 family enhances wound inflammation by targeting LGR4. Journal of Investigative Dermatology 140 (2020): 465–476.e11. 31376385 10.1016/j.jid.2019.07.694

[96] Li D, Le J, Ye J, MiR-361–5p inhibits the Wnt axis via targeting Lgr4 and promotes sepsis-induced myocardial injury. Annals of Clinical & Laboratory Science 52 (2022): 927–937. 36564072

